# Generating Stable Knockout Zebrafish Lines by Deleting Large Chromosomal Fragments Using Multiple gRNAs

**DOI:** 10.1534/g3.119.401035

**Published:** 2020-01-08

**Authors:** Brian H. Kim, GuangJun Zhang

**Affiliations:** * Department of Comparative Pathobiology,; † Purdue University Center for Cancer Research,; ‡ Purdue Institute for Inflammation, Immunology and Infectious Diseases (PI4D), and; § Purdue Institute for Integrative Neuroscience; Purdue University, West Lafayette, Indiana 47907

**Keywords:** CRISPR-Cas9, large knockout, *rnf185*, *rnf215*, *smarca2*, zebrafish

## Abstract

The CRISPR (clustered regularly interspaced short palindromic repeats) and Cas9 (CRISPR associated protein 9) system has been successfully adopted as a versatile genetic tool for functional manipulations, due to its convenience and effectiveness. Genetics lesions induced by single guide RNA (gRNA) are usually small indel (insertion-deletion) DNA mutations. The impact of this type of CRISPR-induced DNA mutation on the coded mRNA transcription processing and protein translation can be complex. Unexpected or unknown transcripts, generated through alternative splicing, may impede the generation of successful loss-of-function mutants. To create null or null-like loss-of-function mutant zebrafish, we employed simultaneous multiple gRNA injection into single-cell stage embryos. We demonstrated that DNA composed of multiple exons, up to 78kb in length, can be deleted in the *smarca2* gene locus. Additionally, two different genes (*rnf185* and *rnf215*) were successfully mutated in F_1_ fish with multiple exon deletions using this multiplex gRNA injection strategy. We expect this approach will be useful for knock-out studies in zebrafish and other vertebrate organisms, especially when the phenotype of a single gRNA-induced mutant is not clear.

The CRISPR (clustered regularly interspaced short palindromic repeats) and Cas9 (CRISPR-associated protein 9) system can induce double-strand breaks in a DNA sequence-specific manner, and generate indel (insertion-deletion) mutations after non-homologous end-joining repair. Thus, it was rapidly implemented into modern biological research since the first successful demonstration in human cell lines due to its simplicity and easy use (Cong *et al.* 2013; Mali *et al.* 2013). Zinc finger nuclease and TALEN (transcription activator-like effector nucleases) are currently being phased out, due to the convenience, simplicity, and efficiency of CRISPR-Cas9, which frequently induce indel mutations around PAM sequences. Originally, only model organisms such as fruit fly, nematodes, mice, and zebrafish (Ma and Liu 2015) were used for mechanistic and evolutionary research, but with CRISPR, other non-model organisms such as axolotl and lamprey have been extended for these interests (Square *et al.* 2015; Flowers *et al.* 2014; Martin *et al.* 2016).

Zebrafish are a powerful model system for vertebrate embryogenesis and human diseases (Dooley and Zon 2000; Lieschke and Currie 2007). They have many advantages, such as a large number of offspring, rapid external development, early transparent embryogenesis, tractable genetics, and relatively low cost compared to murine models. They have been extensively used in developmental studies, and to study a variety of human diseases including cancers. CRISPR has been successfully adopted in the zebrafish research community and has become a cornerstone in many zebrafish research laboratories (Jao *et al.* 2013; Liu *et al.* 2019; Hwang *et al.* 2013). However, the size of these indel genetic lesions induced by CRISPR is usually small (*e.g.*, deletion and/or insertion of a few nucleotides). Thus, altered mRNA processing and other unexpected transcripts may impede the successful rate of CRISPR generating zebrafish null mutants (Tuladhar *et al.* 2019; Anderson *et al.* 2017).

To definitively generate null or null-like loss-of-function zebrafish mutants, multiple exons or whole coding regions ideally should be deleted, but this approach is relatively challenging technically. It has been demonstrated that TALEN can remove up to 1Mb in zebrafish (Xiao *et al.* 2013; Ignatius *et al.* 2018). In this same paper, CRISPR was able to delete 1,423bps within the mir17a-mir92a region in mixed injected fish embryos (Xiao *et al.* 2013). Although TALEN can be effective for the purpose of large deletions, the tedious effector assembly makes it less efficient compared to the ease of CRISPR guide RNA (gRNA) synthesis. Therefore, generating large genomic deletions with CRISPR is the more convenient choice. The feasibility of this method was also demonstrated in a mouse knockout model (Zhang *et al.* 2015).

Here, we report a convenient way of creating 3 zebrafish gene mutant lines with large genomic deletions using the CRISPR-Cas9 system. We successfully created deletions up to 78kbp, as well as simultaneously mutating two genes in a single injection. We expect this approach will be useful for future zebrafish knock-out studies, especially when there is no evident phenotype associated with indel mutations induced by a single gRNA.

## Materials and Methods

### Zebrafish strains and husbandry

Our zebrafish were raised and maintained following the procedures described in the zebrafish book (Westerfield 2000). All experiments were carried out according to the protocols approved by *PACUC* (Purdue Animal Care and Use Committee). The Purdue animal housing facility is an AAALAC-approved animal facility. The wild type line used in this study is of the TAB (also called Tübingen/AB, Tub/AB, or Tu/AB) background.

### Bioinformatics, CRISPR design, and gRNA synthesis

Zebrafish gene coding and transcript information were based on current zebrafish genome annotation (GRCZ11) in Ensembl. CHOPCHOP was employed for gRNA design (Montague *et al.* 2014). For gene-specific oligonucleotides, SP6 promoter sequence was added before the CRISPR RNA sequence followed by an overlap adaptor, which is complementary to the 5′ end of an 80bp constant oligonucleotide according to the published protocol (Gagnon *et al.* 2014). All the oligonucleotides (Supplementary Table 1) were synthesized by IDT (Integrated DNA Technologies). Double-strand DNAs were created by T4 DNA polymerase (New England Biolabs Inc.) after annealing both gene-specific and constant oligonucleotides. *In vitro* transcription was employed for gRNA synthesis using HiScribe SP6 RNA Synthesis Kit (New England Biolabs Inc.). Usually, >10µg RNA can be generated per reaction within 2-4 hr at 37°. All the gRNAs were then purified using a Zymo RNA concentrator-5 kit (Zymo Research) or MEGAclear Transcription Clean-Up Kit (TheromFisher) following manufacturer’s instructions. PCR primers upstream and downstream of the gRNA targeting sites for T7E1 assays were designed in ApE program using default settings and synthesized by IDT. All the PCR primers were optimized using gradient PCR (55-70°) with wildtype genomic DNAs. ORF (open reading frame) analyses were performed in cBioPortal (Cerami *et al.* 2012) and SMART (Simple Modular Architecture Research Tool) (Schultz *et al.* 2000).

### Zebrafish embryo microinjection

Zebrafish embryo microinjections were performed as we described previously (Silic and Zhang 2018). Briefly, adult fish were separated overnight in a fish mating tank by a divider, and 1-cell stage fish embryos were collected in the early morning of the second day immediately before injection. An injection solution was prepared as following: 25ng/µl for each gRNA; 20ng/µl Cas9 Protein (PNA Bio Inc); 0.01% Phenol Red (Sigma). For each embryo, 2nL solution was injected. Dead and deformed fish embryos were removed and healthy ones were raised to adult fish as F_0_ founders.

### Genomic DNA isolation, PCR, and T7 endonuclease 1 (T7E1) assay

Genomic DNAs (gDNA) were isolated by the Hotshot method (Truett *et al.* 2000). For fish embryos, 20 to 30 embryos were pooled 1 day after microinjection. Briefly, each pooled fish embryo was incubated in 100µL 50 mM NaOH at 95° for 1 hr in a thermocycler and then neutralized with 10µL 1M Tris-HCl buffer pH 8.0. For adult fish, gDNAs were isolated with the same method from caudal fin clip instead of whole fish embryos.

For each gRNA, PCRs with flanking primers (Supplementary Table 1) were performed with Taq polymerase at the optimized PCR conditions from gradient PCR tests. Generally, the annealing temperature ranged from 55-70°. To detect large deletions, forward primers from earlier exons and reverse primers from the later exons were used for PCR. The annealing temperature was decided by the overlap range of annealing temperatures of each forward and reverse primers for both targeted exons. Electrophoresis was used for checking PCR results on 1.5–2% agarose gels in sodium boric acid buffer (Brody and Kern 2004).

Guide RNAs’ mutagenesis efficiency was estimated by T7E1 assays as published with some modifications (Jao *et al.* 2013). Briefly, each PCR product was denatured by incubating in 95° for 10 min and then renatured to facilitate heteroduplex formation in NEB buffer 2 (New England Biolabs Inc.) (95°-85°, -2°/s, 85°, 1 min; 85°-75°,-0.3°/s, 75°, 1 min; 75°-65°,-0.3°/s, 65°, 1 min; 65°-55°,-0.3°/s, 55°, 1 min; 55°-45°,-0.3°/s, 45°, 1 min; 45°-35°,-0.3°/s, 35°, 1 min; 35°-25°,-0.3°/s, 25°, 1 min; 12°, hold). We did not perform gel purification, as the PCRs yielded clear target bands. For each reaction, 0.5μL T7E1 enzyme (5units, New England Biolabs Inc.) was added and then incubated at 37° for 1 hr in a thermocycler. Once the incubation is done, the samples were immediately examined on a 2% agarose gel.

The germline transmission rate of mutations induced by each gRNA was assessed by numbers of F1 embryos carrying a mutation of interest from the cross of F_0_ founders with wildtype. A pool of F_1_ zebrafish embryos was harvested for gDNA preparation (Truett *et al.* 2000). After PCR and T7E1 assay for each gRNA, the transmission rate was measured by intensity of both wildtype and mutated DNA bands according to previous reports (Guschin *et al.* 2010). Briefly, the gel images of T7E1 were quantified with ImageJ. Fraction cleaved was calculated as the total signal associated with the cleaved peaks divided by the sum of the signal of the cleaved bands and the un-cleaved band (wildtype). Fraction cleaved was then used to calculate the germline transmission rate using the equation, $100\times (1-{\left(1-fraction\;cleaved\right)}^{\frac{1}{2}})$.

### Gene cloning and plasmid preparation for sequencing

Adult F_1_ fish with mutations detected by PCR were sequenced to confirm DNA sequence changes of the targeted genes. The gDNA sequences around the targeted regions were amplified using primers for T7E1 assays by high fidelity FlashPfu DNA Polymerase (Tonk Bioscience). PCR products were purified with NucleoSpin Gel and PCR Clean-Up (Takara Bio) according to its manufactory manual. The purified PCR products were ligated with the linearized pJet1.2 vector by T4 DNA ligase from CloneJET PCR Cloning Kit (Thermo Scientific). The ligation reaction was transformed into Top10 *E. coli* by heat shock. Single isolated colonies were chosen for plasmid mini-preparation and Bgl II endonuclease (New England Biolabs Inc.) diagnosis since the vector has two Bgl II cutting sites at both ends. Three positive plasmid clones were sequenced at the Purdue Genomics Core Facility by T7 sequencing primer.

### Data availability

Reagents are available upon request. The authors affirm that all data necessary for confirming the conclusions of the article are present within the article, figures, and tables. Supplemental material available at figshare: https://doi.org/10.25387/g3.11541960.

## Results

### Zebrafish smarca2 small and large gene mutations from multiple gRNAs targeting the same gene

**Figure 1:**
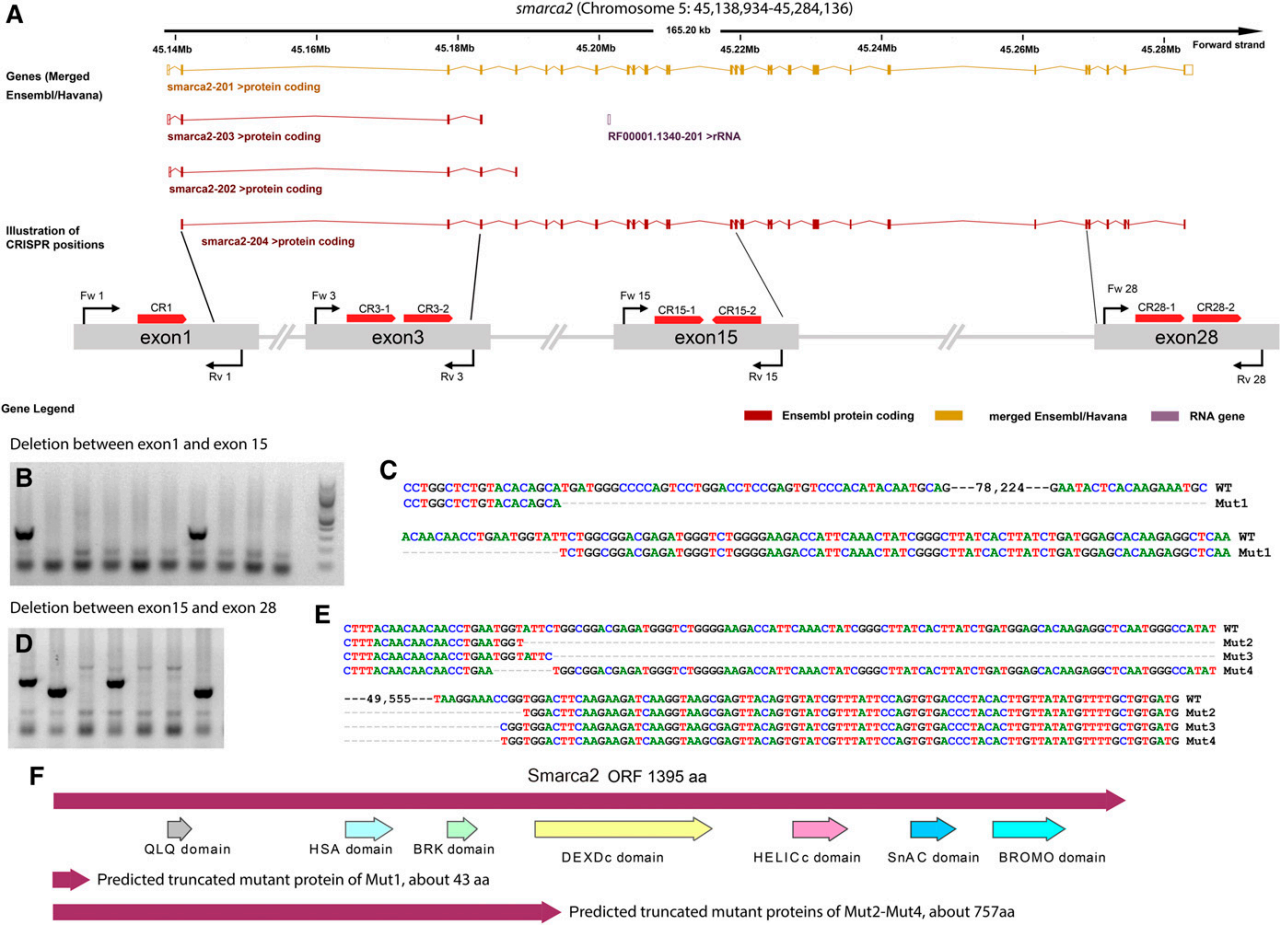
Multiple gRNAs targeting *smarca2* exons induced LKO deletions in zebrafish. (A) Schematic diagram of gRNAs’ targeting *smarca2* exons based on Ensembl annotation. Red bars with arrows indicate the positions of gRNAs against the *smarca2* coding DNA. Red arrows match the gRNA directions. The lengths of illustrated exons are not proportional to the real size of corresponding exons. Black lines and arrows represent primers and their directions. The exon number is based on the longest transcript, *smarca2-204*. Note: Some of the exons may look like one exon on the graph due to their small size and close proximity. (B) Representatives of positive adult F_1_ fish by PCR with primers Fw 1 and Rv 15. No PCR products in wildtype due to the large size of amplicon. PCR works if there is a deletion between exon 1 and exon 15. Each lane is corresponding to an F_1_ adult fish. (C) Large deletion (78,288bp) between exon 1 and exon15 was confirmed by Sanger sequencing. 78,224bp is the size of the omitted region including the dashes. (D) Representatives of positive mutants identified by PCR between exon 15 and 28 with primers Fw 15 and Rv 28. Three different sized PCR bands were identified. Each lane is corresponding to an F_1_ adult fish. (E) Three large deletions (Mut2-Mut4) between exon 15 and exon 28 from different fish were confirmed by Sanger sequencing. Each line represents a type of mutation. 49,555bp is the size of the omitted region including the dashes. (F) Predicted zebrafish Smarca2 wildtype and mutant proteins based on the mutation positions. Protein domains were based on cBioPortal and SMART annotations. Only wildtype protein from the longest transcript (*smarca2-204*) is shown here. Mutant protein sizes were calculated by the ORF finder.

Mutations from a single gRNA are usually small indel genetic changes resulting from double-stranded DNA break repair via non-homologous end-joining mechanism. Whether these DNA mutations have an evident impact on protein translation depends on RNA splicing, RNA nonsense-mediated decay, mRNA misregulation, and other factors (Mou *et al.* 2017; Anderson *et al.* 2017; El-Brolosy *et al.* 2019; Ma *et al.* 2019; Tuladhar *et al.* 2019). We have found several CRISPR-induced zebrafish mutants within the early exons of a few genes that do not have any apparent phenotypes (G. Zhang, unpublished data). Thus, it is difficult to distinguish whether this phenomenon is caused by the incomplete loss-of-function of the small indel mutations, or if the gene is biologically dispensable. This is further complicated given there are only limited available commercial antibodies for zebrafish proteins. One possible solution is to delete all or most of the coding exons of the gene of interest. Especially when there are multiple transcripts coded by a gene locus such as the *smarca2* gene (a.k.a. BRM, Figure 1), it could be difficult to target all the transcripts by a single gRNA. Here, we first chose *smarca2* as an example, since it is a core component of SWI/SNF chromatin remodeler complex (important for embryonic development and cancer). The zebrafish s*marca2* gene is located on chromosome 5. We designed 7 gRNAs to target different exons of the zebrafish *smarca2* gene (Figure 1A), and simultaneously injected these 7 gRNAs into the cytoplasm of 1-cell stage zebrafish embryos: 1 gRNA targeting exon1 and 2 gRNAs targeting each of exons 3, 15, and 28. To examine the individual gRNA efficiency, we performed T7E1 assays on pooled 1-day old injected fish embryos. Indeed, we detected the expected sizes of cleaved DNA bands (Supplementary Figure 1) on agarose gels, suggesting most of these gRNAs are indeed functional although there are some noise bands likely from genetic polymorphism of the PCR amplified region.

The remainder of F_0_ injected fish embryos were raised to adulthood. We randomly selected 11 adult F_0_ founders, and crossed them with wildtype fish for generating F_1_ embryos. From each founder, 20-30 fish embryos were pooled for T7E1 assays to estimate germline transmission rate of mutations induced by each gRNA (Table 1). The rest of the F_1_ embryos were raised to adulthood for further large knockout (LKOs) screening by PCR. As there are two different gRNAs in the targeted *smarca2* exons (3, 15 and 28), small knockouts (SKOs) can occur if two gRNAs worked simultaneously on the same exon. To demonstrate this kind of SKO, individual fins were collected from F_1_ adult fish for genomic DNA. PCR was performed to identify small knock-out mutations between 2 nearby gRNA target sites within each exon (Figure 1A). Nine fish were identified as SKOs within exon 15, and 4 fish with SKOs were identified within exon 28. However, we could not identify any SKOs within exon 3 (Table 3).

**Table 1:** The F_0_ to F_1_ transmission rates of induced mutations by the *smarca2* gene gRNAs

F_0_ founder	CR1 %	CR3–1 %	CR3–2 %	CR15–1 %	CR15–2 %	CR28–1 %	CR28–2 %	1-15 LKO	15-28 LKO	# F_1_ adults analyzed	# F_1_ adults with an LKO deletion	% F_1_ adults with an LKO deletion
**1**	4.95			7.36			17.19		YES	8	1	12.50
**2**	8.16	1.37		6.26			3.61		YES	8	4	50
**3**	10.98	2.80		6.86	2.61		23.19			21		
**4**	7.09						12.50			16		
**5**	6.00			16.63	14.42					20		
**6**	4.39			4.87			3.92	YES		19	3	15.80
**7**	3.73	0.78								0		
**8**	3.18	1.08		1.20						19		
**9**	4.62	0.86		9.04	3.23					26		
**10**	2.12	1.90					0.23			0		
**11**	2.66	1.05		1.05			0.16			17		

**Note:** Empty space indicates that no or uncertain activities detected. LKO: large knockout. The percentage of mutant alleles at each gRNA target site were measured by analyzing T7E1 results from pools of F_1_ embryos, and the percentage of LKO deletion rate was calculated based on F_1_ adult fish.

**Table 3:** Identification of deletion mutations in F_1_ adults

Gene	Forward primer	Reverse primer	Expected PCR size bp	Mutation type	Distance bp	# F_1_ adults with an LKO deletion	# F_1_ adults analyzed	% F_1_ adults with an LKO deletion
***smarca2***	Fw 1	Rv 3	340-430	—	—	0	154	0
	Fw 1	Rv 15	∼310	LKO	78, 224	3	154	1.95
	Fw 1	Rv 28	250-320	—	–	0	154	0
	Fw 15	Rv 28	260-410	LKO	49,644	5	154	3.25
	Fw 3	Rv 3	∼340	—	—	0	154	0
	Fw 15	Rv 15	∼240	SKO	77	11	154	7.14
	Fw 28	Rv 28	∼280	SKO	66	4	154	0.26
***rnf185***	Fw 2	Rv 4	∼240	LKO	3,366	2	48	4.2
***rnf215***	Fw 6	Rv 7	∼320	LKO	1,037	3	48	6.25

**Note:** SKO: small knockout; LKO, large knockout.

Our above data indicated that it is likely that multiple gRNAs can work simultaneously on the same chromosome. Thus, large chromosomal deletions between *smarca2* exons may also be present in adult F_1_ fish. We reasoned that LKOs between exon 15 and exon 28 are most likely since these two exons contain SKO mutations. Besides, we have omitted exon 3 because there were no SKO mutations detected. Therefore, we have tested all the possible combinations between, exon 1 and exon 15, exon 15 and exon 28, and exon 1 and exon 28. The forward primers of earlier exons and the reverse primers of later exons were used for PCR screening to identify LKO mutants. As expected, we were able to identify three different fish with LKO deletion (Figure 1B) between exon 1 and 15 out of 154 adult F_1_ fish from the 9 different F_0_ founders (Tables 1 and 3). In addition, 5 out of 154 fish were also identified to have LKO mutations between exon 15 and 28 (Figure 1D) from different F_0_ founder fish (Tables 1 and 3). Unfortunately, we could not find any fish that had LKO mutations from exon 1 to exon 28. Overall, about 5% of the total number of F_1_ fish contained LKO mutations from the injection of multiple CRIPSRs (Table 3). To further confirm these LKO mutations, we cloned and sequenced the PCR products. Sequencing results confirmed deletions occurred between two gRNA target sites: Mut1 (78,288bp), Mut2 (49,660bp), Mut3 (49,644bp), and Mut4 (49,528bp). (Figure 1 C & E). ORF analysis suggests truncated proteins may result from these LKOs (Figure 1F).

### Simultaneous large-deletion mutations of two genes on the same chromosome from a single injection

**Figure 2:**
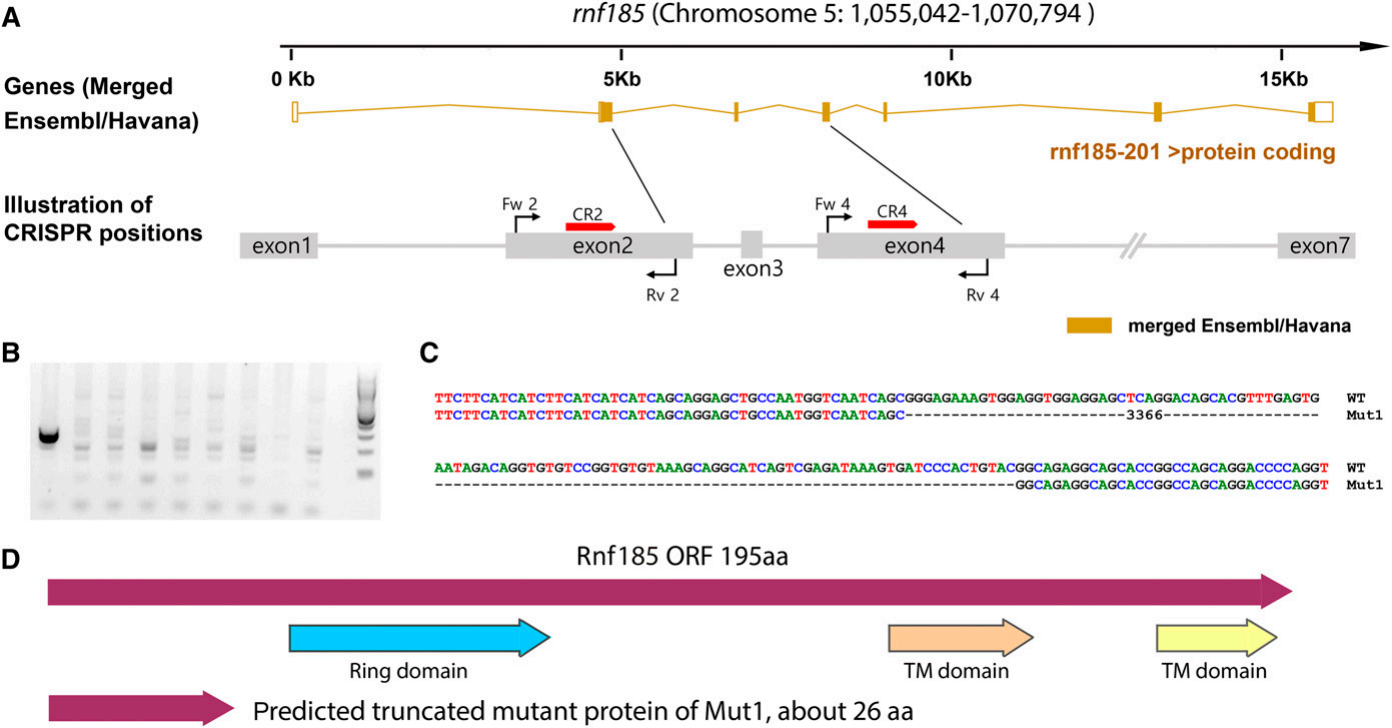
LKO mutations induced by multiple gRNAs targeting the *rnf185* gene in zebrafish. (A) Illustration of gRNAs that are against *rnf185* exon 2 and exon 4 based on Ensemble annotation. Red bars with arrows indicate the positions of gRNAs against the *rnf185* coding DNA. Red arrows match the gRNA directions. The lengths of illustrated exons are not proportional to the real size of corresponding exons. Black lines and arrows represent primers and their directions. (B) Representatives of positive adult F_1_ fish by PCR with primers (Fw 2 and Rv 4) that bind to exons 2 and 4 respectively. Among the 48 fish, 2 were identified to have large deletions between exon 2 and exon 4 (also see Table 1). Each lane is corresponding to an F_1_ adult fish. (C) The exon 2 - exon 4 deletion (3,366bp) was confirmed by Sanger sequencing. 3,366bp is the size of the omitted region including the dashes. (D) Predicted zebrafish Rnf185 wildtype and mutant protein based on the mutation positions. Protein domains were based on cBioPortal and SMART annotations. Mutant protein sizes were calculated by the ORF finder.

**Figure 3:**
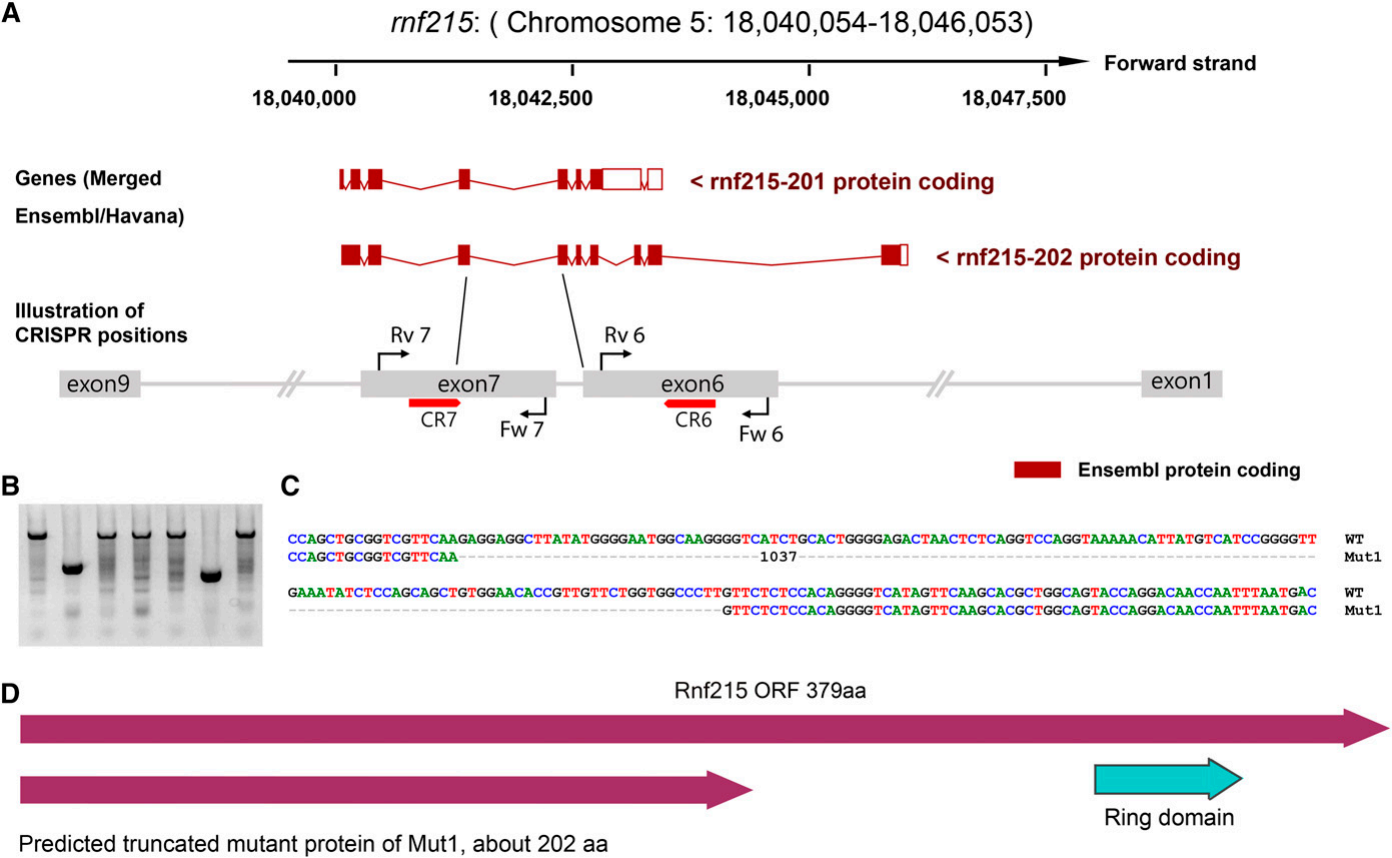
LKO mutations induced by multiple gRNAs targeting the *rnf215* gene in zebrafish. Illustration of gRNAs that are against *rnf215* exon 6 and exon 7 based on Ensemble annotation. Red bars with arrows indicate the positions of gRNAs against the *rnf215* coding DNA. Red arrows match the gRNA directions. The lengths of illustrated exons are not proportional to the real size of corresponding exons. Black lines and red arrows represent primers and their directions. (B) Representatives of positive adult F_1_ fish by PCR with primers Fw 6 and Rv 7 that bind to exon 6 and exon 7, respectively. The top band was amplified from wildtype and the lower small band was amplified from the LKO mutant. Among the 48 fish, 3 fish were identified to have large deletions between exon 6 and exon 7 (also see Table 1). Each lane is corresponding to an F_1_ adult fish. (C) The exon 6 – exon 7 deletion (1,037bp) was confirmed by Sanger sequencing. 1,037bp is the size of the omitted region including the dashes. (D) Predicted zebrafish Rnf215 wildtype and mutant proteins based on the mutation positions. Protein domains were based on cBioPortal and SMART annotations. Only wildtype protein from the longest transcript is shown here. Mutant protein sizes were calculated by ORF finder.

Due to the successful creation of LKOs in the zebrafish *samarca2* gene, we tested the possibility of simultaneously targeting two genes, *rnf185* and *rnf215*, which both are located on zebrafish chromosome 5, but well separated (∼18Mb). As these two genes are relatively small, we chose to target exon 2 and exon 4 of *rnf185* (Figure 2A), and exon 6 and exon 7 of *rnf215* (Figure 3A) by designing 1 gRNA for each exon. We then simultaneously injected all 4 gRNAs into 1-cell stage zebrafish embryos, and validated the effectiveness of the gRNAs (Supplementary Figure 2) by T7E1 assays on a portion of mixed injected fish embryos (20-30). We then raised the rest of these injected fish embryos to adulthood as F_0_ adult candidate founders. Nine F_0_ adult founders were randomly selected and the germline transmission rate of each gRNA induced mutation was evaluated by T7E1 assay on the mixed F1 embryos from the outcross with wildtype fish (Table 2). The rest of the F_1_ embryos from the same crosses were raised to adulthood as F_1_ fish for further LKO screening. As the expected deletions for these two genes are relatively smaller compared to the *smarca2* gene, we randomly selected 48 F_1_ adult fish from the 9 F_0_ founders (Table 2). Adult F_1_ fish were screened for the presence of LKO deletion mutations for each gene by two independent PCRs using forward primers of earlier exons and reverse primers of later exons. As a result, 2 fish were found with LKO mutations between exon 2 and exon 4 in *rnf185* and 3 fish between exon 6 and exon 7 in *rnf215* out of 48 fish screened (Figures 2-3, Table 3). Interestingly, we identified a fish with LKOs in both genes, suggesting that it is possible to simultaneously target two genes in a single germline cell. Similar to the *smarca2* LKO mutant rate, the overall efficiency was 4% for *rnf185*, and 6% for *rnf215* (Table 3). To further validate the LKO mutations, we performed PCR by pairing the early and late exon primers and then cloned the products for sequencing. Deletions of >3kb between exon 2 and exon 4 in *rnf185* (Figure 2C), and >1kb between exon 6 and exon 7 in *rnf215* were confirmed (Figure 3C). ORF analysis suggests truncated proteins resulted from these LKOs (Figure 2D & 3D).

**Table 2:** The F_0_ to F_1_ transmission rates of induced mutations by *rnf185 and rnf215* gRNAs

*rnf185* F_0_ founder	CR2 %	CR4 %	LKO	# F_1_ adults analyzed	# F_1_ adults with an LKO deletion	% F_1_ adults with an LKO deletion
**1**	9.67	16.90		5		
**2**	9.08	11.08		5		
**3**	15.56	26.76		5		
**4**	9.13	12.30	Yes	5	1	20
**5**	15.57	20.24		5		
**6**	3.19	25.36	Yes	5	1	20
**7**	18.31	27.49		5		
**8**	3.84	6.75		5		
**9**	25.65	19.06		8		

*rnf215* F_0_ founder	CR6 %	CR7 %	LKO	# F_1_ adults analyzed	# F_1_ adults with an LKO deletion	% F_1_ adults with an LKO deletion
**1**	3.48			5		
**2**	2.21	8.90	Yes	5	1	20
**3**	1.69	9.59		5		
**4**	1.25	4.11		5		
**5**	1.12			5		
**6**	0.93		Yes	5	1	20
**7**	3.01	7.37	Yes	5	1	20
**8**	0.98	5.96		5		
**9**	2.43	8.39		8		

**Note:** Empty space indicates that no or uncertain activities detected. LKO: large knockout.

The percentage of mutant alleles at each gRNA target site were measured by analyzing T7E1 results from pools of F_1_ embryos, and the percentage of LKO deletion rate was calculated based on F_1_ adult fish.

## Discussion

CRISPR has become an effective reverse genetic tool for zebrafish and other model organisms. Injecting multiple gRNAs simultaneously has been demonstrated to be more effective in mutagenizing zebrafish (Wu *et al.* 2018). Similarly, tRNA-based multiplex gRNA combined with Tol2 transposon has been reported for creating knockout zebrafish (Shiraki and Kawakami 2018). However, this has not been used for creating adult fish lines with LKOs yet, although there is a report with mouse models (Zhang *et al.* 2015). Additionally, a deletion of 1,423bps within the mir17a-mir92a region was reported in zebrafish embryos (Xiao *et al.* 2013). This ∼1.5kb deletion is still relatively small compared to the ∼1Mb deletion using TALEN in the same report, and this deletion was found in the injected F_0_ pooled fish embryos. Here, we demonstrate that LKOs up to 78kb can be created in adult F_1_ zebrafish by co-injection of multiple gRNAs that target different exons of the same gene. Moreover, two different genes on the same chromosome can be targeted simultaneously as exemplified by the *rnf185* and *rnf215* LKO zebrafish. Based on our data, these mutations can be identified in 5% of F_1_ fish. If our F_0_ founder fish were screened by PCR on mixed F_1_ fish embryos, the positive rate of LKOs could be further improved.

Due to the complex impacts of CRISPR-induced DNA mutations on mRNA transcription, splicing, and protein translation, generating indel mutations is not always an effective way to study loss-of-function phenotypes (Anderson *et al.* 2017; Tuladhar *et al.* 2019; Mou *et al.* 2017). In our laboratory, there are several cases where no obvious phenotypes can be observed in CRISPR generated zebrafish with various indel mutations *(kcnj10a*, *kank1a*, *kank1b*, *mdm1*, *etc*. unpublished data G. Zhang). Given the limited availability of zebrafish protein antibodies, it is difficult to distinguish whether single gRNA induced mutations do not lead to complete loss-of-function, or the targeted genes are simply not essential for zebrafish. The latter could be the case, since zebrafish contain paralogous genes, due to bony fish whole-genome duplication (Meyer and Van de Peer 2005; Hoegg *et al.* 2004). In the case of LKOs, most of the key coding exons are deleted, and this is more likely to generate severe loss-of-function mutants, especially when there are multiple RNA transcripts coded or alternative splicing events from the same DNA region. Thus, this approach is useful to solve the uncertainties surrounding a CRISPR-generated zebrafish without an obvious or strong phenotype. One caveat to this approach is there are potentially other coding elements (*e.g.*, miRNAs) within the targeted regions. For example, the zebrafish *omga* gene is located within the 35^th^ intron of the *nf1b* gene. Generally, co-deletion can be avoided if the genome reference is well annotated, however, this is not always the case. Another caveat is nonsense mutations caused by reading frame shift and RNA nonsense-mediated decay. Depending on the protein nature of the targeted gene, mutants can be simply null, dominant-negative, or constant-active. For example, N-terminal truncated Smarca2 may be dominant-negative since the N-terminal contains helicase and bromo domains. Thus, the LKO and indel mutants may provide us a variety of animal models to study targeted gene functions due to mutations that could lead to partial loss-of-function, null, or dominant-negative proteins. In addition, this approach is not only good for making null mutants but also can be an effective tool for studying gene promoters and other *cis*-regulatory DNA elements. For example, the upstream and downstream sequence of the start codon of *smarca*2 gene can be deleted for temporal and spatial expression change. Such gene expression changes can be examined with *in situ* hybridization or RT-PCR on zebrafish embryos or adult tissues.

This multiple gRNA based LKO method is relatively simple, and the directions of the gRNAs for Cas9 may not be important for generating LKOs, as both the same-orientation and opposite-orientation worked well in our experiments. Based on our experience, it seems that one effective gRNA per exon is good enough for LKO generation, but multiple gRNAs against the same DNA region may increase the cleavage rate. We did not thoroughly search for off-target mutations, but it should not be a major issue due to the following reasons: 1. All gRNAs with a single hit in the zebrafish genome were selected. 2. LKOs are a relatively low-frequency event (∼5%) and co-existence of rare off-target mutations is even less probable. 3. Out-crossing with wildtype fish for fish mutant line generation will also eliminate potential off-targets if they indeed exist. If the off-target mutation is key, Cas9 nickase could be another choice, though we did not examine it in this work.

Multiple gRNA cocktail injection into zebrafish embryos has been demonstrated for effective gene disruption in F_0_ fish embryos, and 4-gRNA sets have been computed for 21,386 genes (Wu *et al.* 2018). Interestingly, they indeed reported a low rate of small-sized site-spanning deletion for the *hand2* and *sox32* genes. They found that the frequency of this type of deletion decreases with increasing distance between gRNA target sites (Wu *et al.* 2018). However, no LKO deletion (>1kb) was reported although each gRNA showed high efficiency in the tested genes. Since generating LKO is not their primary goal, their injection method (yolk *vs.* cytoplasm) and mutation detecting method (high-throughput sequencing *vs.* PCR) may miss LKOs in their fish embryos. It is worth mentioning that, very recently, synthetic crRNA:tracrRNA duplex guide RNA was demonstrated highly efficient in zebrafish embryos (Hoshijima *et al.* 2019), and this could be a better choice for improving the LKO generation efficiency in the future.

Simultaneous multiple gene targeting in single microinjection is very useful for studying functionally related genes or genes in the same pathway. Evolutionarily conserved syntenies on chromosomes usually suggest there could be functional constraints, such as in the cases of CDKN2A and MTAP (Mavrakis *et al.* 2016), and TP53 and EIF5A-ALOX15b (Liu *et al.* 2016). Thus, this multiple gene LKO method could be an effective approach for cancer genetics. For example, *rnf185* and *rnf215* are located on the same chromosome in both human and zebrafish. Both chromosomal regions are underrepresented in human and zebrafish malignant peripheral nerve sheath tumors, suggesting they might be tumor suppressor genes. Our *rnf185* and *rnf215* zebrafish mutants may be used for cancer genetic studies in the future.
